# Estimation of Motion and Respiratory Characteristics during the Meditation Practice Based on Video Analysis

**DOI:** 10.3390/s21113771

**Published:** 2021-05-29

**Authors:** Alexey Kashevnik, Walaa Othman, Igor Ryabchikov, Nikolay Shilov

**Affiliations:** 1 St. Petersburg Federal Research Center of the Russian Academy of Sciences (SPC RAS), 199178 St. Petersburg, Russia; nick@iias.spb.su; 2 Information Technology and Programming Faculty, ITMO University, 197101 St. Petersburg, Russia; walaa.s.othman@gmail.com (W.O.); i.a.ryabchikov@gmail.com (I.R.)

**Keywords:** human activity, movement detection, respiratory rate, meditation evaluation, neural networks

## Abstract

Meditation practice is mental health training. It helps people to reduce stress and suppress negative thoughts. In this paper, we propose a camera-based meditation evaluation system, that helps meditators to improve their performance. We rely on two main criteria to measure the focus: the breathing characteristics (respiratory rate, breathing rhythmicity and stability), and the body movement. We introduce a contactless sensor to measure the respiratory rate based on a smartphone camera by detecting the chest keypoint at each frame, using an optical flow based algorithm to calculate the displacement between frames, filtering and de-noising the chest movement signal, and calculating the number of real peaks in this signal. We also present an approach to detecting the movement of different body parts (head, thorax, shoulders, elbows, wrists, stomach and knees). We have collected a non-annotated dataset for meditation practice videos consists of ninety videos and the annotated dataset consists of eight videos. The non-annotated dataset was categorized into beginner and professional meditators and was used for the development of the algorithm and for tuning the parameters. The annotated dataset was used for evaluation and showed that human activity during meditation practice could be correctly estimated by the presented approach and that the mean absolute error for the respiratory rate is around 1.75 BPM, which can be considered tolerable for the meditation application.

## 1. Introduction

Recently, stress has become the most frequent disease in the world and interferes with human lives everywhere. Together with this, health-improving psycho-physiological exercises and self-regulation practices have gained popularity due to an increase of humans’ desire to explore, protect and expand their own spiritual potential [1,2,3] which changes their attitude to life and reduces stress. At the same time, scientists that study the human brain show the positive effect of psycho-physiological exercises on the human brain [4,5]. In other words, meditation practice helps people to cope with stress and depression.

Studying static meditation is both a simple and, at the same time, complex process. The Mindfulness-Based Stress Reduction (MBSR) [6] technique says that one only needs to concentrate on something (e.g., breathing) and forget about thoughts. When the concentration level becomes high enough, the mind relaxes and the body becomes static with small rhythmic movements related to breathing. However, such concentration is a complex task for modern humans. When one tries to concentrate on breathing, she/he has a lot of thoughts with questions like: (1) Am I implementing the practice in the right way? (2) What am I supposed to feel? (3) How much time is left until the end? and others. As a result, when people try to meditate, they do not know if they do it in the right way or not and quit practicing.

At the moment, there are many applications designed to help people with meditation techniques. Some offer progress metrics estimation that usually reflect the measure of calmness and relaxation of the human body during the meditation. For example, the Zendo app (Available online: https://apps.apple.com/us/app/zendo/id1401032490 (accessed on 28 May 2021)) uses a smartwatch to calculate heart rate metrics. The Muse (Available online: https://choosemuse.com/ (accessed 28 May 2021)) device and app is a portable encephalograph to calculate the metrics of brain activity. The Spire app (Available online: https://www.spirehealth.com/ (accessed on 28 May 2021)) uses a device attached to the belt to measure the movement of the torso, from which breathing metrics are further calculated. The disadvantage of these applications is the need to purchase special devices. In this paper, we propose technology for monitoring human body movement, which can be used to estimate the quality of the meditation process, and which only requires the user to have a smartphone with a video camera.

Recently, a lot of research and development efforts related to vision-based human action evaluation methods [7] have appeared. Computer vision techniques are developing fast and enable the recognition of human actions more precisely. Such works consider the detection of different actions and use different approaches.

In this paper, we present an approach to meditation practice evaluation based on image analysis. We have enhanced our previous research [8,9] on this topic. The scientific novelty of the presented paper is as follows: we propose an evaluation system for meditation practice based on image analysis, a contactless method for measuring the respiratory rate using a mobile phone camera, and a method for body parts movement detection with detection of the direction of movement.

We conduct experiments aimed at approach evaluation that have been implemented with the participation of health care professional. Results have been approved by experts and the respiratory rate algorithm shows good accuracy with a mean absolute error of 1.75 BPM.

Although our main interest is the meditation practice, our algorithms such as the respiratory rate calculation and the movement detection can also be used for monitoring children in kinder gardens, driver’s health monitoring and assistant applications.

The rest of the paper is organized as follows: We present related work in the topic of body movement analysis and respiratory rate calculation in Section 2. We propose our method in Section 3. The method includes human body movement estimation, breathing analysis and body movement analysis algorithms. Then we report an evaluation of the proposed method in Section 4. Discussion and limitations of the approach are presented in Section 4. The conclusion summarizes the paper.

## 2. Related Work

In this section, we present a related research analysis on the topic of human action evaluation. We divided the topic into the following subtopics: body movements identification and respiratory rate calculation.

### 2.1. Body Movement Analysis

The authors of the paper [10] present a posture classification system that analyzes human movements from video sequences. In the proposed system, the sequence of movements is converted into a posture sequence. The body is triangulated into triangular meshes to extract the body keypoints and the centroid context feature. Then, the depth-first search algorithm is applied to the triangulation result to extract the skeletal features of the posture, which is used to calculate the centroid features that characterize the shape of the body or body parts.

The authors of the paper [11] propose a 3D visual system to analyze the movement of body parts during therapeutic procedures. The proposed algorithm requires attaching markers with different colors to certain joints’ locations. The camera is used to detect and track the markers in the video to encode their motion information. The detection process is a search algorithm on the matched shape and color. The angle of the joints is calculated for each frame using the law of cosines, then the difference between angles is used to detect and analyze the movement.

The authors of the paper [12] presented a real-time multi-person motion capture algorithm using multi-view video inputs. In the paper, the authors formulate parsing (human keypoints forming in a single image), matching (establishing the correspondences among different views), and tracking (establishing the correspondences between sequential frames) in a unified graph optimization framework (4D association graph) to address the 2D image space, the 1D viewpoint and the 1D temporal information equally and simultaneously. Then, to solve the graph, the authors introduce the idea of 4D limb bundle parsing based on heuristic searching, followed with limb bundle assembling by proposing a bundle Kruskal’s algorithm. Although this algorithm outperforms all the state-of-the-art algorithms, it is quite expensive since multiple cameras in the scene are required, that is, for the described in the paper experiment, five cameras were used to capture the motion of five persons. The proposed algorithms achieved higher accuracy compared to the state-of-the-art method with an average accuracy of 0.976.

The authors of the papers [13,14] propose neural network-based approach for human body keypoints estimation in images. Human keypoints estimation can be used to detect body movement and classify actions of people [15]. Unlike approaches presented above, this approach utilizes only a single image of a person without additional preparations such as marking. A keypoint detection error is relatively high (for [14] it is 47 MPJPE mm). In action classification, where the magnitude of the movements significantly exceeds the keypoint estimation error (for example, squatting, punching, or passing an object), this error can be tolerated, but for detecting a movement such as a chest motion while breathing, the error is too high.

The authors of the paper [7] present an overview of action evaluation approaches applied to areas such as health care, skill training and sport activity scoring. Many observed approaches introduce a prior stage of estimating human movement in various forms (movement of human body keypoints, optical flow, etc.). These approaches are focused on detecting and evaluating only actions expressed by strong motion, such as walking, climbing stairs, brushing teeth, or fighting. In our work, we consider actions expressed by the slightest movement: breathing, straightening the shoulders, and slouching.

Optical flow can be used to detect the smallest pixel-scale movements in videos. The authors of the paper [16] propose the SelFlow convolutional neural network for optical flow estimation that has been trained on the KITTI [17] and MPI Sintel [18] datasets. The neural network has shown some of the best results among open implementations with an average error less than 2.5px. It was able to detect the smallest movements of objects of various sizes, shapes and homogeneous textures.

### 2.2. Respiratory Rate Calculation

The Respiratory rate (RR) is an important indicator for meditation practice estimation. In this section, we review different approaches and consider the appropriate sensors for RR measurement.

The authors of paper [19] introduce an automated method of measuring the children’s respiratory rate using a pulse oximeter device to obtain the photoplethysmogram (PPG). Then they applied the wavelet transform to calculate the RR. The experiment was conducted on children ages between 18 months and 12 years. The approach is valid for adults as well, but the focus of the research is on children since they tend to move a lot which makes measuring the RR a challenge. The error rate was around one breath per minute.

The authors of [20] propose a method based on the synchronous evaluation of a 3D time-of-flight camera and a microwave interferometric radar sensor for measuring the respiratory rate of neonates. The authors use the 3D camera to obtain 3D point clouds used to calculate the displacement of the moving chest and from it the respiratory rate. The microwave interferometric radar sensor, on the other hand, determines the change in displacement caused by respiration and measure the small superimposed movements due to the heartbeat. The method showed good results for normal respiratory rate with a maximum error of 3 BPM.

In another study [21], the authors propose an ROI detection algorithm based on both temporal and spatial consistency (TSC), which aims to extract the representative characteristics of respiration with fewer computational resources. The proposed algorithm was tested on a dataset of more than 50 hours of sleeping people and showed a good results for breathing rate 0.97 bpm.

The authors of paper [22] present an algorithm for remote respiratory rate recognition. The proposed method extracts the photoplethysmographic signals from facial video images in the visible light spectrum. The algorithm can be divided into three main steps: (1) Signal extraction: which contains the ROI detection (forehead and the face skin) and the PPG signal extraction, (2) preprocessing: contains the filtering and signal modulation techniques, (3) post-processing: contains signal normalization, artifacts reduction using the differentiation of the signal prior to FFT analysis. The algorithm was tested on two datasets and showed good results.

The authors of the paper [23] used videos obtained by placing an adult finger on a mobile phone camera of 19 subjects and recorded using smartphones. The proposed algorithm in the paper is based on smart fusion and empirical mode decomposition method. The smart fusion-based algorithm detects the artifacts using an incremental merge segmenting algorithm, then extracts three respiratory induced variations components and calculates the RR from each of them. The final RR is the average of the calculated RRs. The empirical mode decomposition-based method uses fast Fourier transform to get the spectral power of the imaging PPG signal. The frequency with maximum power was considered to be the heart rate signal. The signal is filtered and decomposed into four intrinsic mode functions and their residual signal. Then, the spectral power of each component is calculated, and the frequency peak with the highest power in the spectral domain is extracted. The frequency peak with the highest power is extracted as the RR estimation after eliminating the peaks located near the heart rate frequency. The mean square error they recorded was around six breathes per minute.

The authors of the paper [24] propose an approach to extract near-continuously respiratory rate using videos of faces recorded by an industrial camera with a spatial resolution of 659 × 494 pixels and 60 fps, and equipped with a 15 mm fixed focal length lens. The data were then processed to derive the video PPG signal. To estimate the RR, three features have been extracted: pulse rate variability, pulse amplitude variability and pulse width variability.

In another study [25], the authors introduce an approach to calculate the respiratory rate from a short video of the subject’s face by measuring the fluctuation in the hue channel in the HSV color space. Since the algorithm completely depends on the face, it will not work well if the forehead is partially or fully covered or if the face detection fails.

The authors of paper [26] introduce an algorithm to estimate the RR during apnea based on multi-spectral data fusion. The respiratory information is extracted from multiple sources (far-infrared and near-infrared cameras). For that, a multi-spectral region of Interest (ROI) localization algorithm has been applied to find the chest and nostrils regions, then localized ROIs are used to extract the thermal airflow signal from the nostril ROI and the respiratory motion signal from both the far-infrared and near-infrared chest ROIs.Then the noise to signal ratio is calculated and used as the weight for the weighted median fusion to calculate the respiratory rate. The mean square error was 1.60 breaths/min.

In general, video-based methods are used to calculate the respiratory rate, detect a region of interest, then some signal processing methods are used to filter and extract the RR. Our method detects the thorax movement, then filters the signal and calculates the peaks as they express the inhales (more detailed information is shown in Section 3.2).

Table 1 summarizes the methods for evaluation of the respiratory rate.

## 3. Method

**Figure 1:**
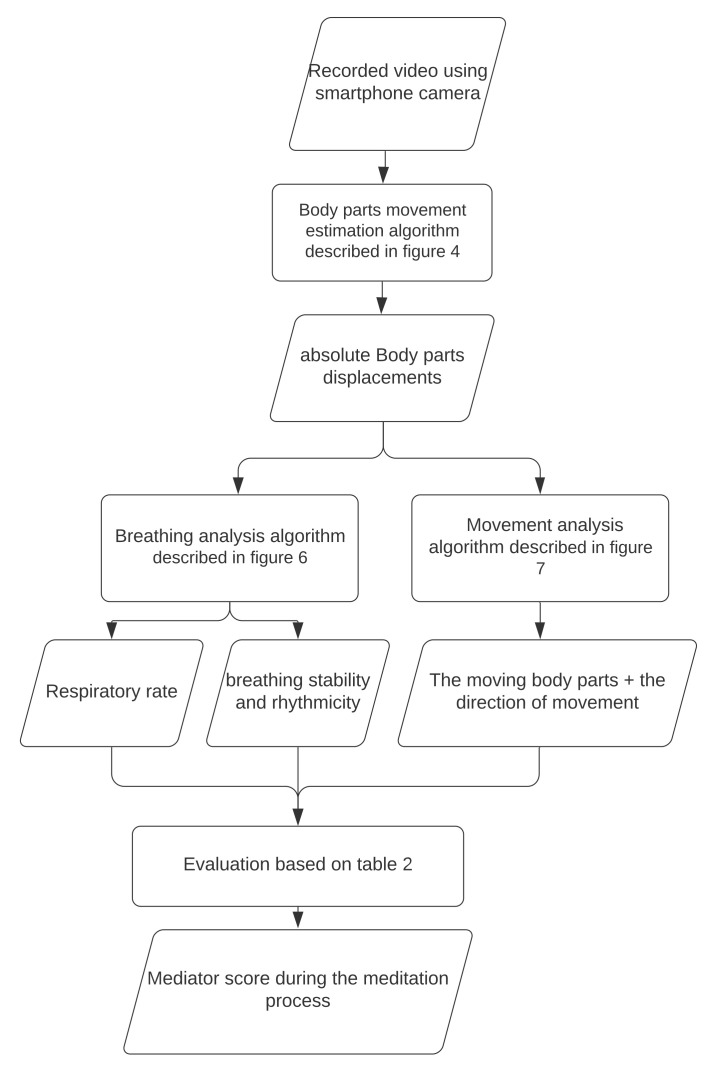
The block diagram of the proposed meditation practice evaluation method.

In the section, we propose a meditation practice evaluation method based on video analysis of the meditator. The block diagram of the proposed method is shown in Figure 1. We describe in detail an algorithm for keypoints detection as well as estimation of body parts movement. Based on such estimation, we propose an algorithm for body movement analysis that allows us to detect bending and to straighten the back, movement of head, body, hands and legs; and an algorithm for breathing analysis that allows us to determine the respiratory rate as well as rhythmicity.

### 3.1. Human Body Movement Estimation

For the purpose of meditation analysis automation, we propose an approach to the movement estimation of the following human body parts: head, thorax, shoulders, elbows, wrists, stomach and knees. By analyzing their movement, we estimate breathing parameters (such as frequency, depth, rhythmicity, etc.) corresponding to a different concentration degree; detect sudden movements arising from the choice of an uncomfortable posture or muscle tension and correlating with the loss of concentration, and detect common mistakes in meditation such as slouching and incorrect performance of movement patterns adopted in a particular meditation technique.

We propose the application of optical flow estimation to measure the movement of the human body. The optical flow is calculated for a pair of frames and represented as a set of two-dimensional vectors of pixel displacement between these two frames. To estimate the optical flow, we use the SelFlow [16] convolutional neural network.

**Figure 2:**
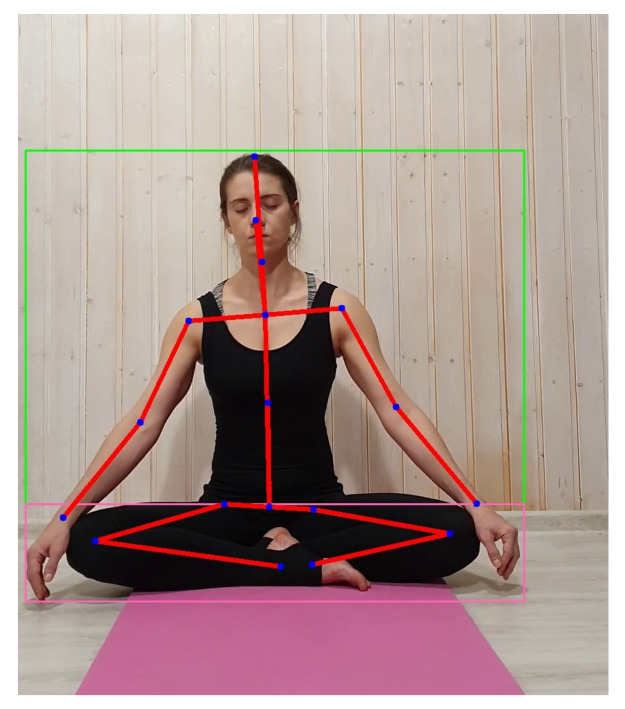
Human body keypoints detection.

For the detection of individual body parts, we propose to use the neural network from the paper [14]. It takes an RGB image as input and returns a set of coordinates of the human body keypoints (see Figure 2). Before estimating keypoints, we use the Mask R-CNN [27] to detect the person’s bounding box in the image. Preliminary detection of the person’s bounding box is necessary to achieve the required accuracy of the keypoints estimation (an image patch is supplied as an input instead of the whole frame), as well as to make sure that the person is present in the frame.

**Figure 3:**
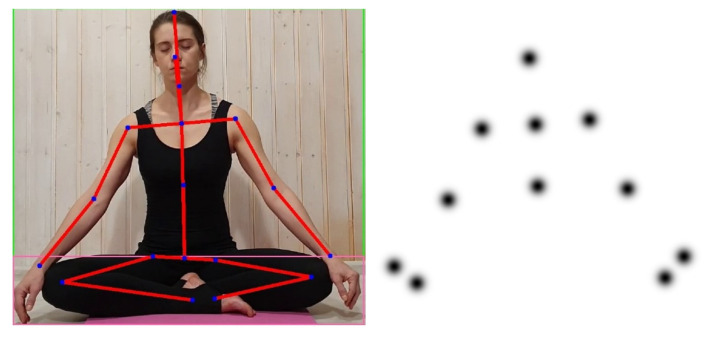
Application of Gaussian function to calculate displacement vectors.

Then, using the obtained optical flow and keypoints coordinates, the absolute displacement vectors are calculated. The projections of these vectors give us the motion graphs for each part of the human body: movement along the *x*-axis (right/left) and movement along the *y*-axis (up/down). To obtain a displacement vector of a keypoint, the weighted sum of the optical flow vectors is calculated. The weights are determined by a Gaussian function (Formula (1)) centered at the corresponding key point (Figure 3). Calculation of the weighted sum is necessary for greater robustness, taking into account the optical flow and keypoints estimation error. Since the displacement vector is measured in pixels, in order to be able to compare the results at different camera resolutions, distances from a person to the camera, and sizes of person’s bodies, the vector is normalized by dividing it by the shoulder width (the distance between the shoulders keypoints in pixels).

(1) $$\frac{1}{\delta^{2}*2\pi }*e^{-\frac{{\left(x-\mu_{kpx}\right)}^{2}+{\left(y-\mu_{kpy}\right)}^{2}}{2\delta^{2}}},$$

where δ - a parameter equal to 10% of the shoulder width; x,y - pixel coordinates; $\mu_{kpx},\mu_{kpy}$—body keypoint coordinates.

**Figure 4:**
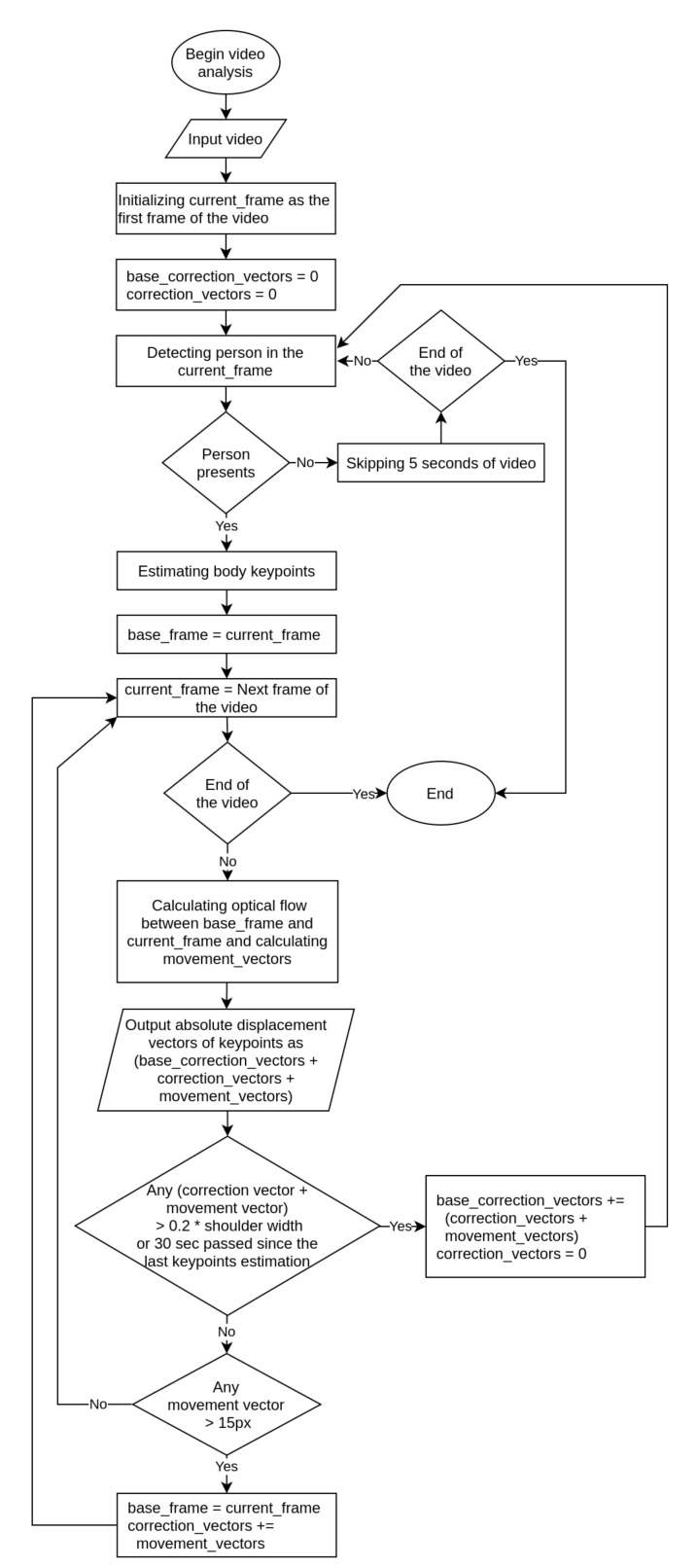
Body parts movement estimation algorithm.

The algorithm for processing a meditation video to obtain the movement graphs of individual human body parts is shown in Figure 4. We assume that the processed video of meditation may not contain a person in the beginning due to the recording set up, the person may temporarily disappear in the middle of recording (for instance, due to camera adjusting) and may not present at the ending of the video. A person may move during the recording—sitting down at the beginning of meditation or changing posture during meditation.

First, we detect a frame containing a person using FasterRCNN neural network. To do so, we examine frames every 5 s until a person is detected. Next, we estimate the human body keypoints, initialize the current frame as the base frame and calculate the optical flow and displacement vectors for each subsequent frame until a displacement vector of any keypoint is greater than 15 pixels. Then we make the current frame the new base frame and add the calculated displacement vectors to the correction vectors. Correction vectors are summed with future displacement vectors to get the absolute displacement from the beginning of the video even after the base frame is changed. Updating the base frame after a significant movement of a body part is required due to the increase in the optical flow estimation error, which can be explained by the decrease in the similarity of corresponding human body parts in frames due to the changes in the surrounding area, changes of shadows and rotation of body parts. At the same time, the estimation of the optical flow using the base frame instead of adjacent frame is implemented in order to minimize the error, which increases with each update of the correction vector. For a small movement (of the order of few pixels) the relative error of displacement vectors estimation can be high. The maximal displacement vector size of 15 pixels was chosen experimentally on our meditation dataset (Available online: https://cais.iias.spb.su/meditation/index.html (accessed on 28 May 2021)).

When the displacement of any keypoint from the initial position becomes greater than 20 percent of the shoulder width we re-estimate all keypoints. We assume that such a significant displacement can lead to incorrect body parts position estimation carried out via optical flow only. In addition, the keypoints re-estimation will be carried out every 30 s in order to reset the accumulated error of body parts position.

If at the next keypoint re-estimation we do not detect a person, we try to detect a person every 5 s of the video, and the interval at which the person was absent will not be taken into account in the movement graphs.

**Figure 5:**
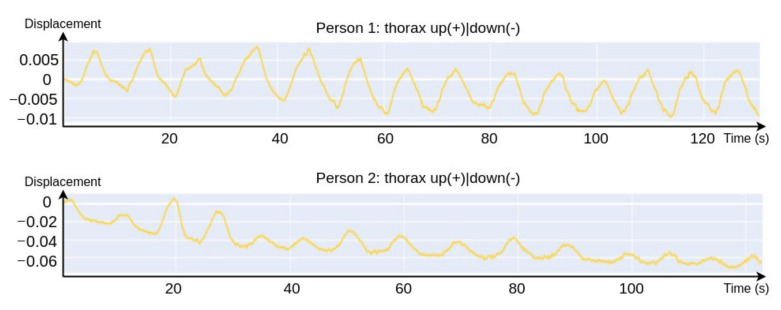
Thorax movement graphs of two people during meditation.

As a result of video processing, we get 22 graphs of absolute displacement (trajectory) of 11 keypoints. Figure 5 shows the result example - thorax movements graphs of two people during meditation. The graphs clearly show the breathing of the people, as well as the gradual slouching of the second person. The human body parts trajectory can be used to classify more complex actions, for example, to classify various everyday activities (playing piano, push ups, tennis swing, etc.) [28] or to detect fights [29].

### 3.2. Breathing Analysis

Breathing analysis is the key feature that defines how good the meditation process is. Deep breath with a low rate is a sign of relaxation. In this section, we discuss the method we use to calculate the respiratory rate, its stability and rhythmicity. After detecting the movement of the thorax using the mentioned above algorithm, we propose to clean data using the rsp_clean function from the neurkit2 library [30]. The function is based on the Zero-crossing algorithm with amplitude threshold proposed in the paper [31]. The cleaning process consists of two steps.

1. Linear detrending: The drifts/fluctuations are removed from the raw signal to make it centered around zero. This can be done by approximating and removing the trend component from the signal;
2. Filtering: We apply a fifth-order 2 Hz low-pass infinite impulse response (IIR) Butterworth filter. We propose to apply a low-cut at 0.05 Hz and a high-cut at 3 Hz filters to preserve respiratory rates between 3 breath per minute and 180 breaths per minute.

After cleaning the data, the respiratory rate is calculated by counting the number of peaks at every minute. For the meditation process, we, besides the respiratory rate, care about other breathing characteristics (breathing stability and breathing rhythmic). By that, we mean whether the breathing is stable and rhythmic or not. To determine this, we rely on the two main characteristics of a wave: the height of a wave (amplitude) and the distance between adjacent crests (wavelength). For the rhythmicity property, we calculate the peak to trough (P-to-T) amplitude at each breath and consider the breath to be rhythmic if the difference between the P-to-T amplitude for all breaths during the meditation process and the median one is less than the experimentally determined threshold, taking into consideration the fact that they should be small since they represent the tolerance. For the stability property, we consider the breath to be stable if the peaks were relatively close to each other (the difference between successive peaks are small) and the same for troughs (the difference between successive troughs are small).

**Figure 6:**
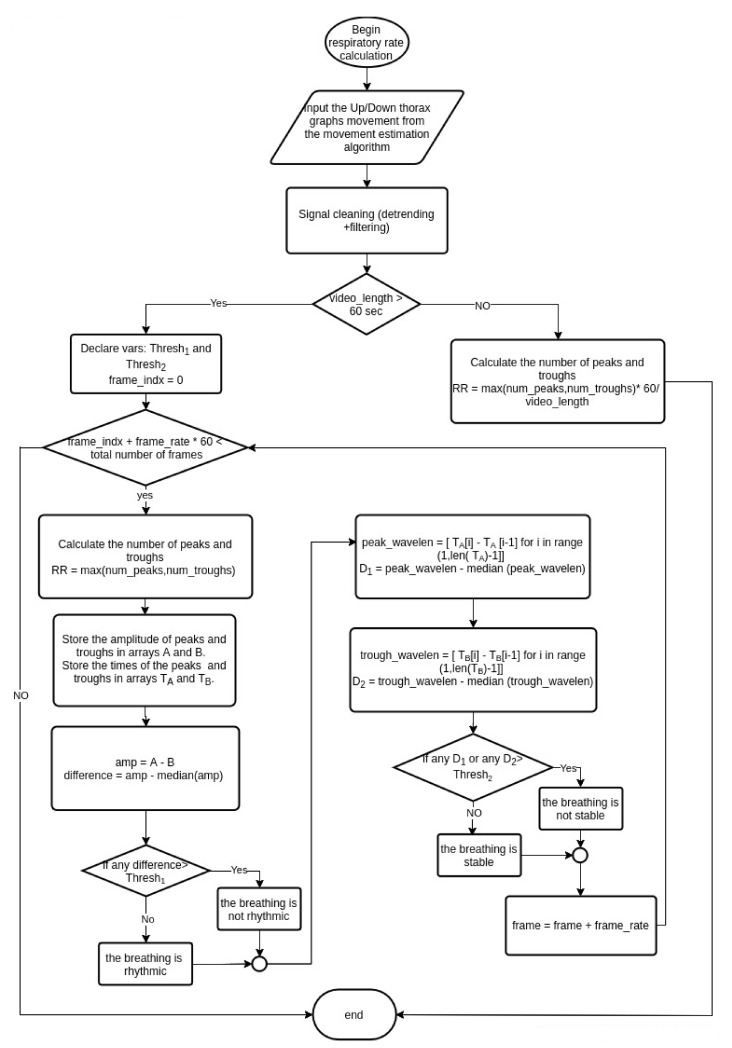
Breathing analysis algorithm.

Figure 6 shows our algorithm for calculating the respiratory rate and evaluating the rhythmicity/stability properties. The input of the algorithm is the movement graph of the thorax (Section 3.1). The input graph is cleaned in two steps: detrending and filtering, as we described earlier in this section. Since the respiratory rate represents how many inhales/exhales are taken per minute, which reflects in thorax movement as the number of trough/peaks in one minute, we defined two cases: when the video is shorter than one minute and when it is longer. If the video is shorter than one minute, we drop the calculations of breathing stability and breathing rhythmic because the period is not enough to evaluate them, and we only calculate the respiratory rate by finding the number of peaks and the number of troughs and take the maximum between them then scale this number by the ratio video length in seconds over 60. If the video is longer than one minute, we declare two thresholds Thresh_1_ and Thresh_2_ to evaluate the stability and rhythmicity of the breathing. These thresholds are determined experimentally. The evaluation and the RR calculation are applied on a time window between the frame_idx and the frame_idx + 60* frame_rate where the frame_idx represents the current frame, and the frame_rate is the number of frames per second. We conduct the experiments with the frame_rate equal five frames per second. For each window, first, the peaks and the troughs are found, their values are stored in arrays A and B, respectively, and the time when they occurred are stored in arrays T_A_ and T_B_ respectively. The RR is then calculated by taking the maximum between the number of peaks and the number of troughs. To evaluate the rhythmicity of the breathing, the amplitude array is calculated by taking the difference in values between the peaks and their corresponding troughs, then we find the median amplitude (median) and consider the breathing rhythmic if all the amplitudes are with the range [median-Thresh_1_, median+Thresh_1_]. For breathing stability, we calculate the wavelength between the adjacent peaks and then between the adjacent troughs. For each, the median is calculated and subtracted from the wavelengths. The difference then is stored in D_1_ and D_2_ arrays for peak wavelengths and trough wavelengths, respectively. The breathing is considered stable if all the elements of D_1_ and D_2_ are less than threshold Thresh_2_.

### 3.3. Body Movement Analysis

**Figure 7:**
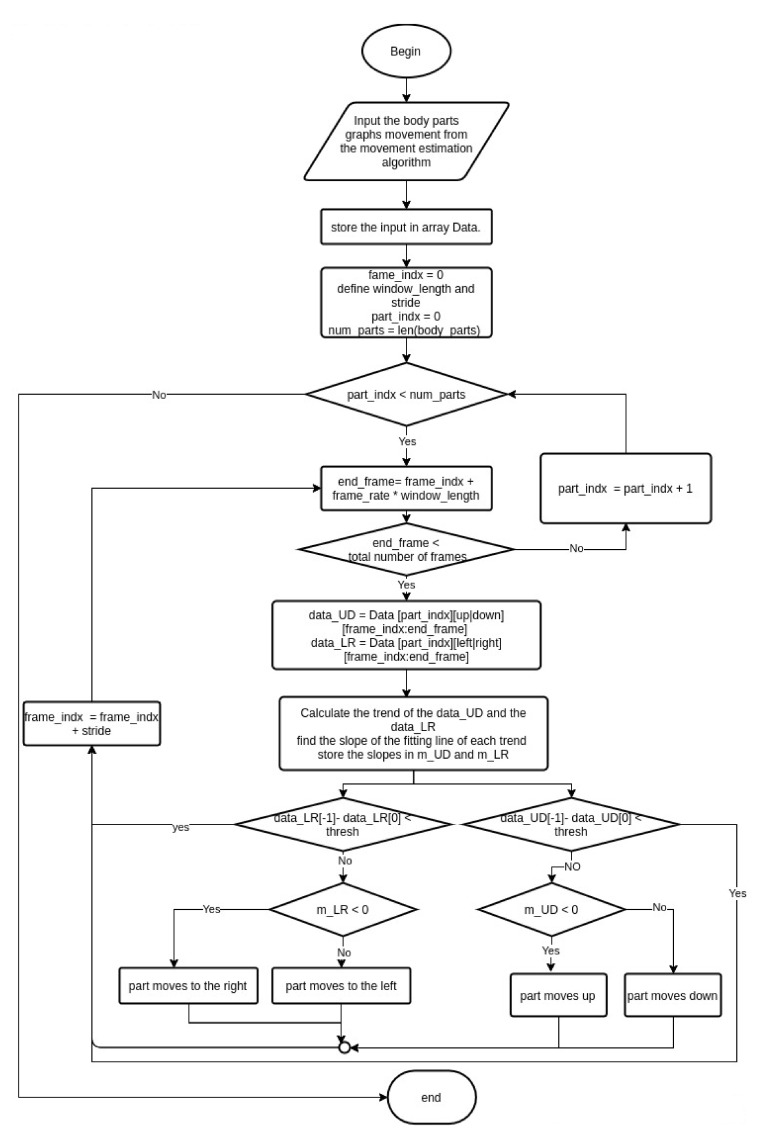
Movement analysis algorithm.

Analyzing the movement of the meditator is mandatory due to its importance in evaluating the meditation process. In this section, we describe the proposed method used to detect the movement and determine its direction. Figure 7 shows our algorithm to calculate the respiratory rate and evaluate the rhythmicity/stability properties.

The input to the algorithm is the keypoints movement graphs discussed in Section 3.1). These graphs represent the motion of the body parts divided into two main categories based on the axis of movement: the up/down and right/left movements. This algorithm evaluates the direction of the meditator movement for every 1.2 s with a shift of 1 s. In other words, we are using a sliding window with a length of 6 frames (1.2 s) and a stride of five frames (1 s). The reason to choose the window length to be 1.2 s is the ability to handle fast movement as well as achieve overlap between successive windows. For each part, the algorithm finds the direction of the movement on the two axes. To detect the direction of the movement, and since the movement is a time series, we decompose the signal on each axis into its main components: trend, seasonality and residual. Then, we find the fitting line of the trend component of the signal and calculate the slope of this line. To prevent the algorithm from detecting the unimportant micro movements, we ignore the movement if the difference between the pose at the final and the initial frames of the window is less than a threshold we defined as 9 millimeters. We calculated the threshold imperially based on manual analysis of videos of professionals as well as beginners in meditation practice.

If the slope is negative, we consider that the direction of movement is to left/down depending on the axis of movement we are evaluating. On the other hand, if the slope is positive, then the direction is to the right/up.

## 4. Evaluation

In this section, we will discuss the results of implementing the mentioned algorithms. The section is organized as follows: firstly, we consider the experiment methodology, then the breathing results, after that the movement analysis results, and, finally, the meditation evaluation.

### 4.1. Experiment Methodology

Our dataset consists of more than 90 videos for 17 different people [8] who vary in age, gender and level of meditation experience. For the breathing analysis, we show the results for four different meditators: three beginners and one professional. The beginner meditators have been chosen according to the movement during the video: high, medium, and low since the movement related to stress affects the respiratory rate. For the rest sections, we show the results for one beginner and one professional meditators.

### 4.2. Breathing

Here, we discuss the results of the breathing analysis for four different people. The RR is calculated for each graph using a sliding window with the length of 300 frames (60 s) and the stride five frames (1 s), which means that the RR calculated for the period [t,t+60] where *t* is the time in seconds t∈[0,T−60] and *T* is the total time of the video. The RR is considered high if it is over ten breaths per second [8].

**Figure 8:**
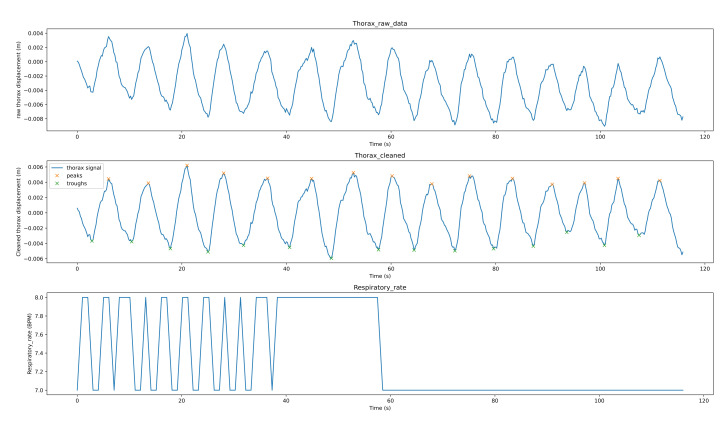
Breathing analysis for a professional meditator.

**Figure 9:**
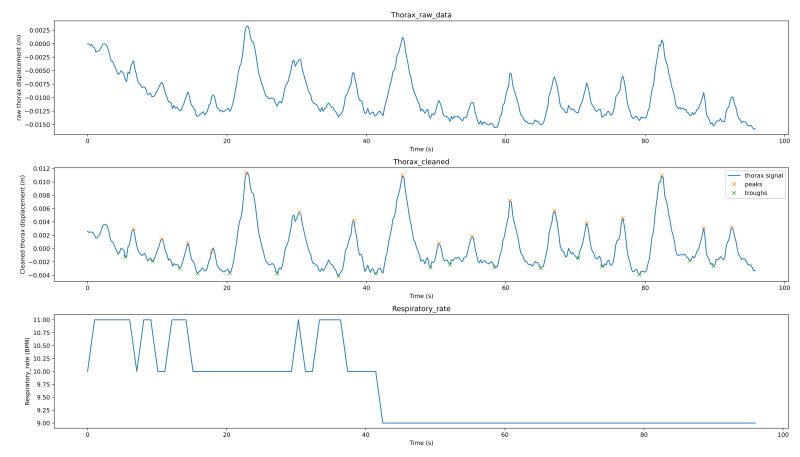
Breathing analysis for a beginner meditator.

**Figure 10:**
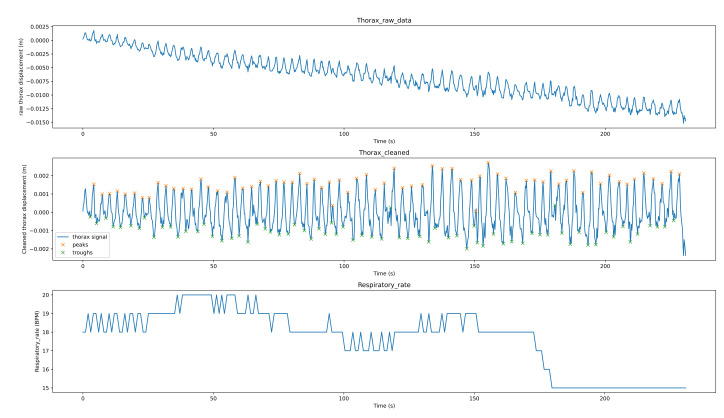
Breathing analysis for a beginner meditator.

**Figure 11:**
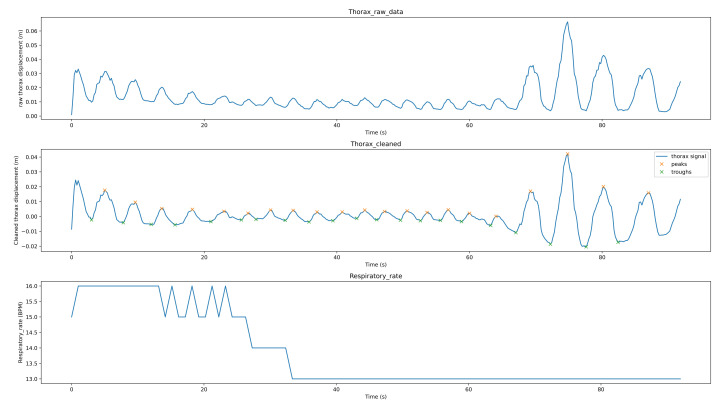
Breathing analysis for a beginner meditator.

Figure 8 shows the breathing analysis for a professional meditator. The first graph is the raw thorax movement signal (the result of the movement estimation algorithm), the second graph is the cleaned signal (after applying filtering and detrending) with the detected peaks (red points) and troughs (green points), while the last graph is the respiratory rate recalculated each second. For this meditator, the breath is both stable and rhythmic. The respiratory rate is low and ranges between 7–8 breaths per minute. Figure 9 shows the breathing analysis for a beginner meditator. As we can see, the RR is between 9–11 (which can be considered a low rate, which means deep breathing). The breathing is not rhythmic nor stable. Figure 10 shows the breathing analysis for another beginner meditator. As we can see, the RR is between 15 and 20 (high), and the breathing is both rhythmic and stable. Figure 11 shows the breathing analysis for another beginner meditator. The RR is between 13 and 16 (high), and the breathing is not rhythmic and not stable.

### 4.3. Movement Analysis

**Figure 12:**
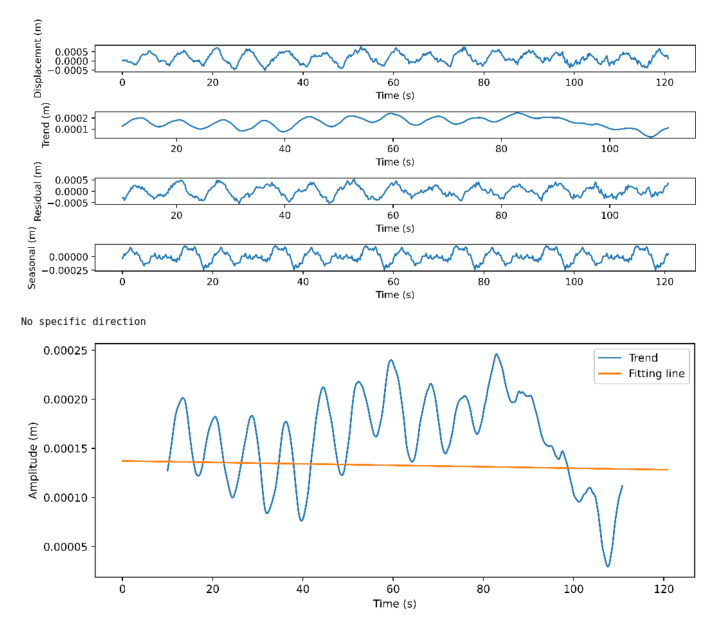
Full body up/down movement analysis for a professional meditator.

Here, we discuss the results of the body movement analysis approach presented in Section 3.3. Although when evaluating the meditator direction of movement, we implement it each second for a period of 1.2 s. Here we first present, for the sake of clarity, the results of applying the approach on two videos with different lengths to evaluate the direction of whole-body movement on the horizontal axis (up/down movement). Figure 12 shows the movement analysis of the full body in the direction up/down for a professional meditator. The figure consists of two sub-figures. The first one shows the raw signal with its main components: the trend, the seasonal, and the residual components, while the second sub-figure shows the trend component in blue and its fitting line in red. Then the direction is calculated based on the slope of the fitting line.

**Figure 13:**
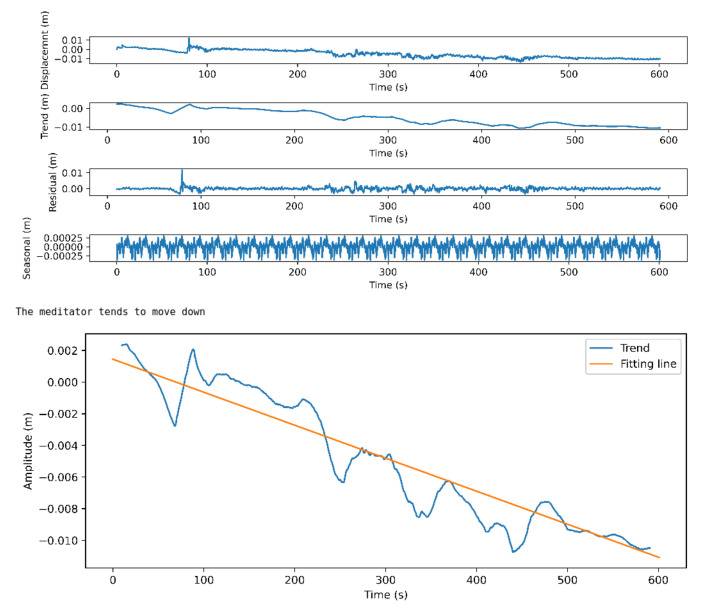
Full body up/down movement analysis for a beginner meditator.

Figure 13 shows the movement analysis of the full body in the direction up/down for a beginner.

**Figure 14:**
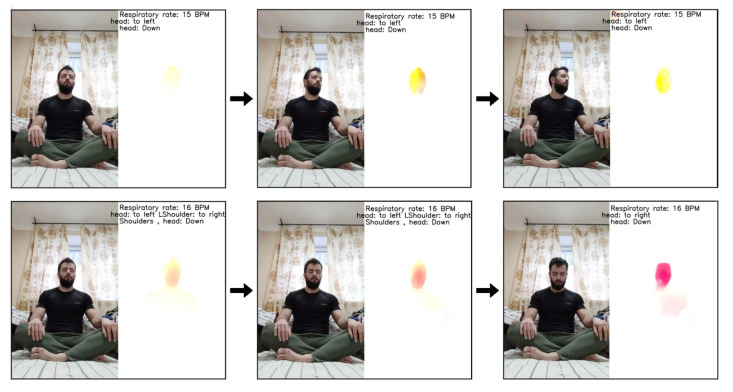
Movement analysis results.

**Figure 15:**
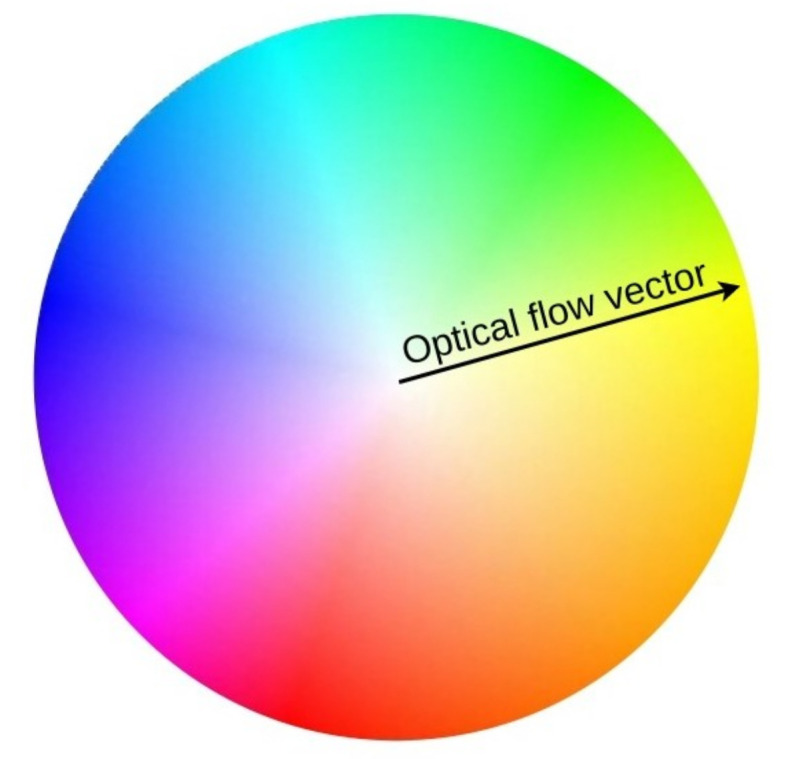
Color wheel that defines the heatmap pixels color.

Figure 14 shows the results of applying the algorithm directly to a video. The figure shows six sequential frames taken with a step of two frames for a beginner meditator. At the right side of every figure, the movement heatmap is shown as well as labels that show the algorithm output. The color of the heatmap pixels is determined by the direction of the optical flow vectors in accordance with the color wheel (Figure 15). Transparency is determined by the magnitude of the vectors. For example, moving the head to the left (from the meditator’s point of view) causes the yellow color in the heatmap, to the right causes the blue, to the down causes red and to the up we will see green-blue.

### 4.4. Meditation Evaluation

In this section, we describe the evaluation of the meditation process that is an enhancement of our previous paper [8]. In this paper, we make the evaluation based on the breathing and movements parameter according to the proposed model (see Table 2).

where:

- Introduction phase: the first 2 min of the whole meditation time;
- Conclusive phase: the last 1–2 min of the whole meditation time;
- Main phase: the residual time period.

**Figure 16:**
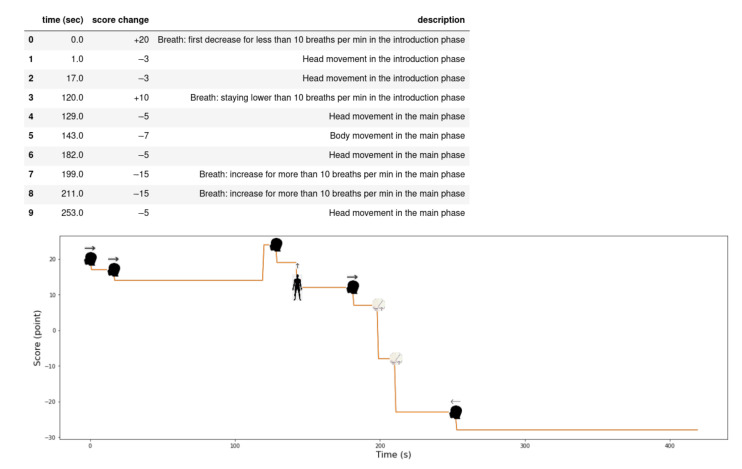
Meditation evaluation for a beginner meditator.

**Figure 17:**
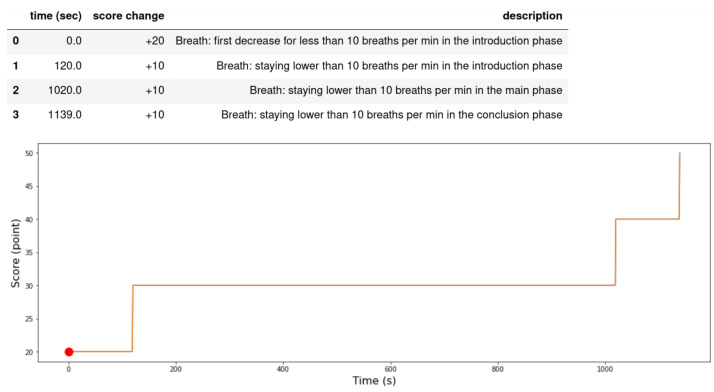
Meditation evaluation for a professional meditator.

The following rules presented in the table we evaluated based on analysis of different meditators behaviour. Figure 16 shows the evaluation of a beginner meditator (the x-axis represents the time in seconds and the y-axis represents the score) with drawings on the graph determine the movement of the body and the head with an arrow that represents the direction of the movement. The reasons the score improved or went down are shown above the figure. Figure 17 shows the scores of a professional meditator.

### 4.5. Mobile Application and Algorithms Evaluation

For algorithm evaluation we created a dataset that consists of 8 videos of men and women of different ages with different respiratory rates as well as with motions (head, hand, leg). The dataset has been recorded by different smartphones. The approach showed good results for the movement detection algorithm (100%), and acceptable results for the respiratory rate with an MAE of 1.75 BPM. The detailed results of the breathing are shown in Table 3. The movement detection algorithm showed an accuracy of 100% in both detecting the body parts movement and the direction of the movement.

**Figure 18:**
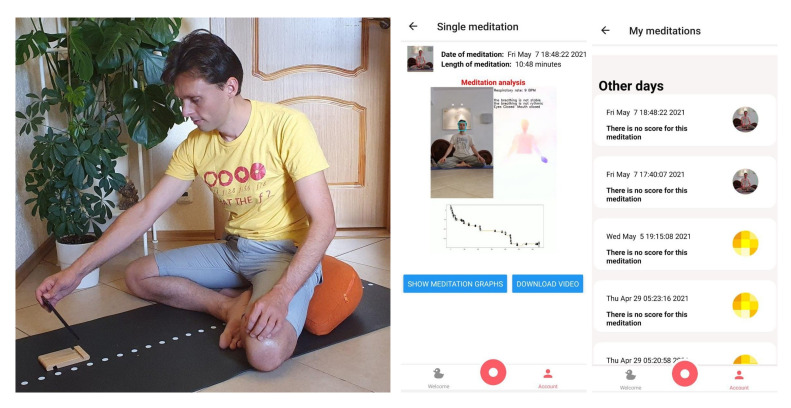
The meditation application interface.

Figure 18 presents how the process of the recording looks like including the smartphone that is use (left side) as well as the mobile application interface for the proposed methods (center and right). Meditators use the app to record a video during the meditation process (as shown on the left of Figure 18). Then the video is sent to the server where the algorithms proposed in the paper are applied. Then the meditator can see the results in the mobile application (see center and right picture in Figure 18).

## 5. Discussion and Future Work

The aim of the presented research is to analyze the quality of performance of psycho-philological exercises, particularly, the static meditation practice, so that a person could realize that she/he is doing the practice in the right way. Whereas the most of the available today applications pursuing the same goal are based on the usage of additional sensors (from fitness wristband to portable encephalographs), the proposed approach makes it possible to use only a smartphone equipped with a camera and connected to the Internet. Another advantage of the proposed approach is that it generates the overall evaluation score what makes it possible for the meditator to compare different meditation practice sessions and track the progress.

Unlike other approaches, the presented approach does not require any specific equipment for evaluating the quality of the meditation practice, which makes it potentially usable by a wide audience. The approach is contactless, the person does not need to place a finger on the camera to measure the respiratory rate. The system is complex and compound of sub-algorithms to collect the movement and respiratory rate information. Besides, timeline-based visualizations and generation of the overall practice score enable practitioners to analyze their performance, understand their own mistakes and track the progress.

The proposed approach has been evaluated using an annotated dataset of eight videos produced by four people. Besides, the approach has been tested through analysis of videos produced by 17 people performing the meditation practices and having different levels of experience. Since no quantitative indicator can be calculated to compare the accuracy of the proposed algorithm, the results of the evaluation have been presented and discussed with skilled meditation experts and teachers (experts) who confirmed that the meditation practices were evaluated correctly enough for self-estimation by regular practitioners.

A smartphone is an essential part of experiment and we have evaluated main requirements it should meet. The main characteristic is the video camera, which should support Full HD video recording. Since videos are recorded with a big size 10 min of meditation takes about 1 GB of memory. So the needed amount of memory should be supported. Processor and RAM do not matter, they only need to support Full HD video recording. The smartphone should have an Internet connection.

Despite the promising results and acceptable accuracy, the approach has some limitations. It relies on a number of parameters that can significantly affect the estimation results. Currently, the only considered possibility to define these parameters is to define some basic values based on common sense and then to adjust these through a discussion of the estimation results with an expert. For the presented experiment, the parameters were defined on the basis of the analysis of ten practitioners with different skills. However, the correct work of the algorithm for the other seven people confirmed by the expert, shows that such chosen parameter values are capable of generalization.

Another limitation is related to the fact that meditation can be performed in a dimmed environment. Though modern smartphones are capable of recording video in poorly lit environments; this potentially can be a problem for the application of optical flow and image analysis techniques. Currently, this issue is a subject of future work.

## 6. Conclusions

In this paper, we presented an approach to evaluating the meditator’s performance during mediation practice. For that, we proposed a camera-based algorithm for calculating the respiratory rate and breathing characteristics like stability and rhythmicity. We also proposed a method for detecting the movement of body parts as well as the direction of the movement. The algorithm takes as an input the video stream of the participants from a smartphone camera.

The methods were evaluated using an annotated dataset consisting of eight videos of different meditators.The mean absolute error for the respiratory rate algorithm is 1.75 BPM. The experiments showed the applicability of the approach and its independence of a particular practitioner.

Future work is aimed at increasing the precision of the approach by involving more participants as well as analyzing the performance of the approach in dimmed conditions since the meditation practices can be carried out in an environment with low light. We are planning to develop methods to detect the state of the eye and mouth of the meditator and to estimate the meditator’s head pose to get more information about his/her state. The case of straightening the back and squaring the shoulders will be detected. We are also planning to try our approach for dynamic meditation practice (e.g., OSHO Dynamic Meditation). We are planning to conduct experiments in OSHO Center in St. Petersburg, Russia. So, we can conclude that further implications of the presented approach can include human state analysis in various circumstances. For example, a similar approach can be used for the analysis of the state of a personal computer operator or a vehicle driver. Analysis of the breathing parameters and movements can be used for the evaluation of the levels of fatigue, concentration or aggressiveness.

## Figures and Tables

**Table 1:** Summary of the respiratory rate methods.

Paper	Used Sensor	Methods and Application	Error
[19]	pulse oximeter device	The RR was calculated by applying wavelet transform on the PPG obtained by the device from children ages between 18 months and 12 years	1 BPM
[20]	3D TOF camera and a microwave interferometric radar	The respiratory rate was calculated from the chest displacement obtained from 3D point clouds collected from 3d camera. The radar measures the small superimposed movements due to the heartbeat	maximum error: 3BPM
[21]	Camera	The ROI detection algorithm based on both temporal and spatial consistency (TSC), which aims to extract the representative characteristics of respiration with fewer computational resources.	MSE: 0.95 BPM
[22]	camera	The proposed method depends on extracting the PPG signal from the forehead and face skin with modulations, filtering, and artifact reduction to extract the respiratory pattern	-
[23]	camera	The PPG signal is obtained using FFT from a video recorded by placing an adult finger on a mobile phone camera	MSE: 6 BPM
[24]	camera	The PPG signal is obtained from the face region	-
[25]	camera	The RR was obtained by measuring the fluctuation in the hue175channel in the HSV color space	-
[26]	far-infrared and near-infrared cameras	The chest and nostrils is extracted using multi-spectral region of Interest localization algorithm, then the thermal airflow signal is extracted from nostril and the respiratory motion signal from chest. The RR was calculated by dusinf the RR from the two ROIs	MSE: 1.6 BPM

**Table 2:** The evaluation parameters and their scores.

Activity Description	Introduction Phase	Main Phase	Conclusive Phase
Breath: first decrease for less than 10 breaths per min	+20	0	0
Breath: increase for more than 10 breaths per min	−5	−15	−10
Breath: staying lower than 10 breaths per min	+10	+10	+10
Head movements	−3	−5	0
Changing the position of lower body	−3	−10	−5
Changing the position of hands	−3	−5	0
Body movement	−3	−7	−2

**Table 3:** The detailed results of the breathing.

Video	Ground Truth	Estimated RR	Accuracy
1	18	16	88%
2	34	31	92%
3	10	9	90%
4	24	22	92%
5	13	12	92%
6	8	7	88%
7	9	8	89%
8	35	32	91%

## Data Availability

Our dataset is available by request on the following web page: https://cais.iias.spb.su/meditation/index.html.
